# Supplementary material for: Sorting at embryonic boundaries requires high heterotypic interfacial tension
Source: Nat Commun. 2017 Jul 31;8:157. doi: 10.1038/s41467-017-00146-x (PMC5537356; doi:10.1038/s41467-017-00146-x)
Supplement: Supplementary file 2 — Supplementary Software 1 [file 41467_2017_146_MOESM2_ESM.zip › PottsModel/SrcPottsModel/doc/mvc/Observer.html]

Observer


JavaScript is disabled on your browser.


Skip navigation links


- Overview
- Package
- Class
- Use
- Tree
- Deprecated
- Index
- Help

- Prev Class
- Next Class

- Frames
- No Frames

- All Classes

- Summary:
- Nested |
- Field |
- Constr |
- Method

- Detail:
- Field |
- Constr |
- Method


mvc

## Interface Observer

- All Known Implementing Classes:
  :   Lattice, PlotPanel, PottsCanvas, PottsFrame, PottsLogger, SnapshotManager, StatisticsManager, StatusBar

  ---

    

  ```
  public interface Observer
  ```

  Interface that can be implemented to provide observer support.

  Author:
  :   Eleyine Zarour

- - ### Method Summary

    All Methods Instance Methods Abstract Methods

    | Modifier and Type | Method and Description |
    | --- | --- |
    | `void` | `update(javax.management.Notification pNotification)` Determines what an observer should do upon notification that the observed object has changed. |

- - ### Method Detail


    - #### update

      ```
      void update(javax.management.Notification pNotification)
      ```

      Determines what an observer should do upon notification that the observed object has changed.

      Parameters:
      :   `pNotification` - : Notification passed by the object being observed.


Skip navigation links


- Overview
- Package
- Class
- Use
- Tree
- Deprecated
- Index
- Help

- Prev Class
- Next Class

- Frames
- No Frames

- All Classes

- Summary:
- Nested |
- Field |
- Constr |
- Method

- Detail:
- Field |
- Constr |
- Method
